# Nucleotide Sequence Variation in the Insulin-Like Growth Factor 1 Gene Affects Growth and Carcass Traits in New Zealand Romney Sheep

**DOI:** 10.1089/dna.2020.6166

**Published:** 2021-02-11

**Authors:** Shaobin Li, Huitong Zhou, Fangfang Zhao, Qian Fang, Jiqing Wang, Xiu Liu, Yuzhu Luo, Jon G.H. Hickford

**Affiliations:** ^1^ Gansu Key Laboratory of Herbivorous Animal Biotechnology, Faculty of Animal Science and Technology, Gansu Agricultural University, Lanzhou, China.; ^2^ International Science and Technology Cooperation Base of Meat Sheep and Meat Cattle Genetic Improvement in Northwest of China, Gansu Agricultural University, Lanzhou, China.; ^3^ Gene-Marker Laboratory, Faculty of Agricultural and Life Sciences, Lincoln University, Lincoln, New Zealand.

**Keywords:** insulin-like growth factor 1 gene, sheep, polymorphism, carcass, growth

## Abstract

Insulin-like growth factor 1 (IGF1) is a mediator of the effects of growth hormone and polymorphism in the IGF1 gene (*IGF1*) is reported to affect fat deposition in some livestock species. In this study, nucleotide sequence variation in three regions of ovine *IGF1* (part of the 5′ flanking region, the exon 3 region, and the exon 4 region) was investigated in 848 New Zealand Romney lambs using PCR-single strand conformation polymorphism (SSCP) analyses to ascertain if single nucleotide polymorphisms (SNPs) existed. Six SNPs were identified across these three regions. The effect of the sequence variation in the exon 3 and exon 4 regions on growth and carcass traits were investigated. One of the PCR-SSCP sequence variants in the exon 3 region was associated with variation in hot carcass weight, carcass fat depth at the 12th rib measured using video imaging and the percentage proportion of leg lean meat, whereas the other was associated with variation in growth rate to weaning. No associations were detected for the other gene regions analyzed. The results suggest that polymorphism in exon 3 of ovine *IGF1* has potential for use as a gene-marker for some carcass and growth traits.

## Introduction

Insulin-like growth factor 1 (IGF1) is encoded by the IGF1 gene (*IGF1*) (Jansen *et al.*, 1983; Hoppener *et al.*, 1985). It has “non-suppressible insulin-like activity” (Salmon and Daughaday, 1957) and is a primary mediator of the effects of growth hormone. Growth hormone is synthesized in the anterior pituitary gland and released into the blood stream. It stimulates the liver to produce IGF1, which can then fuel body growth by having growth-promoting effects on almost every cell in the body, while also regulating cellular DNA synthesis (Yakar *et al.*, 2002).

In mammals, *IGFI* is composed of six exons separated by five introns, and it spans >80 kb (Rotwein, 2012). The nucleotide sequence and length of exons 1–4 are conserved across species, whereas exons 5 and 6 are more variable. Exons 1 and 2 determine the class of the protein and encode the signal peptide for cellular localization after translation, whereas exons 3 and 4 primarily encode the mature IGF1 peptide. This ultimately becomes the receptor-binding ligand (Rotwein, 2012).

Polymorphism of *IGF1* has been reported to affect growth and production traits in a number of livestock species. It has been reported that a single nucleotide polymorphism (SNP) in the promoter of *IGF1* affects fat deposition and carcass merit traits in hybrid Angus and Charolais beef cattle (Islam *et al.*, 2009); and in dairy cattle, SNPs in *IGF1* have been associated with growth-related traits, carcass fat, milk production, and milk fatty acid traits (Mullen *et al.*, 2011; Li *et al.*, 2016). In pigs polymorphism of *IGFI* is associated with final body weight, average daily gain and back-fat thickness (Niu *et al.*, 2013), whereas in goats an *IGF1* SNP affects growth traits (Zhang *et al.*, 2008).

There have been a number of studies investigating the effects of *IGF1* polymorphism on growth and production traits in different sheep breeds, but the results do at times conflict. Some researchers have reported polymorphism in the 5′-flanking region of *IGF1* associated with growth traits in Baluchi (Tahmoorespur *et al.*, 2009), Makui (Hajihosseinlo *et al.*, 2013) and Makooei sheep (Negahdary *et al.*, 2013), but no associations between *IGF1* polymorphism and growth traits were detected in Indian Madras Red sheep (Ramasamy, 2018), Polish Pomeranina coarse-wool sheep (Proskura and Szewczuk, 2014), Zandi sheep (Nazari *et al.*, 2016) and Baluchi sheep (Gholibeikifard *et al.*, 2013). With Colored Polish Merino sheep, polymorphism in the 5′-flanking region of *IGF1* not only affected growth and body size, but also affects carcass and meat quality traits (Grochowska *et al.*, 2017). SNPs in *IGF1* intron 1 were found to be associated with a number of carcass traits in Santa Ines sheep, including internal carcass length, rump girth, rib yield and neck weight (Meira *et al.*, 2019).

Despite the interest in ovine *IGF1*, research has tended to focus on SNPs in the 5′ flanking region and introns. Little is known about whether nucleotide sequence variation in the other regions of *IGF1* has an effect on growth and carcass traits, and whether the effect exists in common breeds in major sheep-farming countries.

In this study, we used PCR-single strand conformation polymorphism (PCR-SSCP) analyses to search for SNPs in the *IGF1* 5′ flanking region, and in the exon 3 and 4 region that encode the IGF1 mature peptide in New Zealand (NZ) Romney sheep, the most popular sheep breed in NZ. The effect of the PCR-SSCP haplotypes on growth and carcass traits was subsequently investigated.

## Materials and Methods

All research involving animals was carried out in accordance with the Animal Welfare Act 1999 (NZ Government) and the collection of sheep blood drops by nicking sheep ears is covered by Section 7.5 Animal Identification of the Animal Welfare (Sheep and Beef Cattle) Code of Welfare 2010, which is a code of welfare issued under the Animal Welfare Act 1999 (NZ Government).

### Sheep investigated and data collection

Eight hundred forty-eight NZ Romney lambs, the progeny of 19 unrelated industry-sourced rams that were part of a progeny test on a commercial farm, were investigated. The gender, birth weight, birth rank (i.e., whether they were a single, twin, or triplet), and rearing rank were recorded for each lamb. All the lambs were weaned at ∼90 days of age, weighed, and separated based on gender into two mobs. The preweaning growth rate of the lambs was calculated as the average daily weight gain (grams/day) from birth to weaning.

As most of the female lambs were kept as ewe replacements for the larger commercial base flock, the draft weight and carcass data were only available from male lambs and a small number of cull ewe lambs. Lambs weighing >37 kg were first drafted for slaughter at around 16 weeks of age, with a second draft at ∼20 weeks of age. All remaining male lambs were slaughtered at ∼24 weeks of age. Draft weight and draft age were recorded for each lamb.

Hot carcass weights (HCWs) were measured directly on the processing chain (Alliance Food Limited, Smithfield, Timaru, NZ), which is the weight in kilograms of the carcass minus the head, gut, and pelt. Video image analysis (VIAScan; Sastek, Australia), developed by Meat and Livestock Australia and described by Hopkins *et al.* (2004), was used to estimate the following carcass traits: lean meat yield (expressed as a percentage of HCW) in the shoulder (shoulder yield), loin (loin yield) and leg (leg yield), and total yield (the sum of the shoulder, loin and leg yields for any given carcass), and V-GR (a VIAScan assessment of subcutaneous fat depth near the 12th rib). To describe the distribution of lean meat across the carcass, the proportion of total yield of shoulder, loin, or leg was also recorded, this being the yield of the specific part of the carcass divided by the total yield and expressed as a percentage.

At tailing, blood samples from all these sheep were collected onto TFN paper (Munktell Filter AB, Sweden) by nicking the lamb's ears and genomic DNA was then purified for PCR analysis using a two-step procedure described by Zhou *et al.* (2006).

### PCR primers and amplification of ovine IGF1

Three pairs of PCR primers were designed manually to amplify a portion of the 5′-flanking region, the entirety of the exon 3 region (including parts of its flanking introns) and the exon 4 region (including parts of its flanking introns) of *IGF1* (Table 1). The PCR primers were chosen based on analysis of the ovine genome sequence Oar_v4.0 NC_019475.2, and checked for suitability as primers using DNAMAN (version 5.2.10; Lynnon BioSoft, Vaudreuil, Canada). The primers were synthesized by Integrated DNA Technologies (Coralville, IA).

**Table 1.** PCR Primers and PCR-Single Strand Conformation Polymorphism Conditions for the Analysis of Ovine *IGF1*

Region amplified	Primer sequence (5′-3′)	Predicted amplicon size	PCR annealing temperature	SSCP condition
5′ flanking	CAGTTGGCTTTACAGCTCAG	340 bp	60°C	25°C, 270 V, 15 h
	CATCTGCTAATACACCTTACC			
Exon 3	CTGCTCAGAGGTCACTCAC	452 bp	62°C	31°C, 250 V, 19 h
	GCTGAAACACTAGGCTCGC			
Exon 4	GACTGCTGGAGATATACTGG	389 bp	62°C	28°C, 250 V, 15 h
	CTGGTGGGCTTACCTTCTG			

IGF1, insulin-like growth factor 1; SSCP, single strand conformation polymorphism.

The PCR amplifications were carried out using S1000 thermal cyclers (Bio-Rad, Hercules, CA), and were performed in a 15-μL reaction containing the purified genomic DNA on a 1.2-mm punch of the TFN paper, 0.5 U Taq DNA polymerase (Qiagen, Hilden, Germany), 0.25 μM of each primer, 2.5 mM Mg^2+^, 150 μM of each dNTP (Bioline, London, UK) and 1 × the reaction buffer supplied with the enzyme. The thermal profile for amplification consisted of 2 min at 94°C, followed by 35 cycles of 30 s at 94°C, 30 s at the annealing temperatures shown in Table 1, and 30 s at 72°C; with a final extension of 5 min at 72°C.

### Screening for sequence variation and sequencing of PCR-SSCP haplotypes

The PCR amplicons were screened for nucleotide sequence variation using SSCP analysis. A 0.7-μL aliquot of each amplicon was mixed with 7 μL of loading dye (98% formamide, 10 mM EDTA, 0.025% bromophenol blue, and 0.025% xylene-cyanol). After denaturation at 95°C for 5 min, the samples were cooled on wet ice and then loaded on 16 cm × 18 cm, 14% acrylamide:bisacrylamide (37.5:1) (Bio-Rad) gels. Electrophoresis was performed using Protean II xi cells (Bio-Rad) in 0.5 × TBE buffer, and the electrophoretic conditions shown in Table 1. Gels were silver stained according to the method of Byun *et al.* (2009).

The PCR amplicons identified as homozygous by SSCP analysis were directly sequenced at the Lincoln University Sequencing Facility, NZ. Sequence alignments, translations, and comparisons were carried out using DNAMAN. The SNPs that were revealed were named using the nomenclature described online and aligned to GenBank NC_019475.2 (*Ovis aries* breed Texel chromosome 18), Oar_v4.0.

### Statistical analyses

There were some missing data, and sheep with incomplete records were removed from some analyses. Sample numbers, therefore, vary in different analyses. Statistical analyses were performed using Minitab version 17 (Minitab, Inc., State College, PA).

Two types of General Linear Mixed-Effects Models (GLMMs) were used to ascertain the effect of *IGF1* genotype on the measured traits. The first models ascertained the effect of the presence/absence (recorded as 1 and 0) of the PCR-SSCP variant sequences on the measured traits. The second models were pairwise comparisons between genotypes using a Tukey test with Bonferroni corrections. The core model for these analyses was Yijklm = μ + S_i_ + G_j_ + B_k_ + D_l_ + V_m_ + e_ijklm_, where Y_ijklm_ is the trait measured on each animal (birth weight, etc.), μ is the mean for the trait, S_i_ is the random effect of sire, G_j_ is the effect of gender, B_k_ is the effect of birth weight, birth rank, or rearing rank, D_l_ is draft age, V_m_ is the fixed effect of genotype or the presence/absence of each variant, and eijklm is the random residual error. For the birth weight GLMM, gender and birth rank were fitted into the models as fixed factors, but with the growth to weaning GLMMs, gender and rearing rank were fitted into the models. For carcass and yield traits, gender, birth weight, and draft age were fitted into the models as covariates. Only main effects were tested, and associations were considered significant at the 5% level.

## Results

### Nucleotide sequence variation in ovine IGF1

**FIG. 1.**
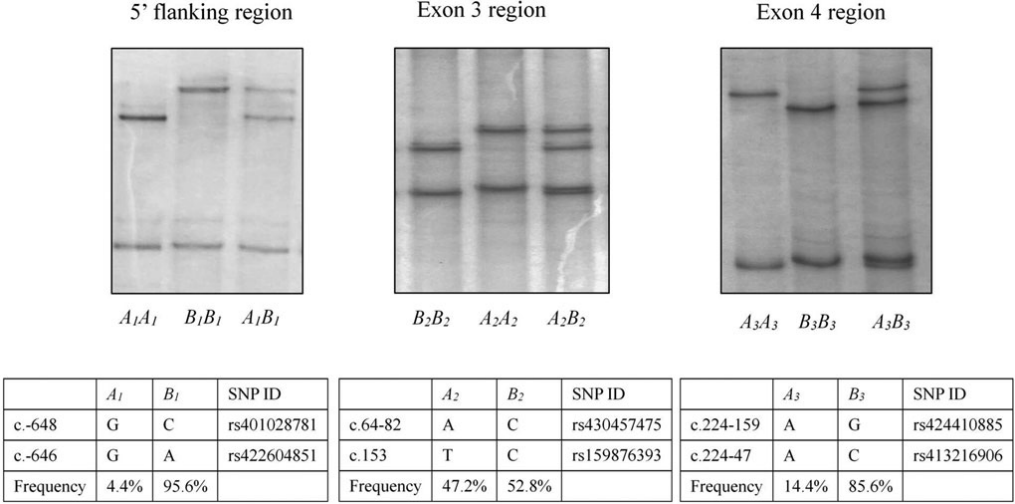
Sequence variation in ovine *IGF1*. Different sequences identified in three regions of *IGF1* using by PCR-SSCP analysis and DNA sequencing. The nucleotide substitutions in these sequences are illustrated, together with their frequencies in the 848 sheep investigated. *IGF1*, insulin-like growth factor 1; PCR-SSCP, PCR-single strand conformation polymorphism.

Two unique PCR-SSCP banding patterns were detected in each region of ovine *IGF1*, with either one or a combination of two banding patterns observed for each sheep (Fig. 1). DNA sequencing revealed that these PCR-SSCP patterns represented six unique sequences of *IGF1*. The six sequences have been deposited into GenBank with accession numbers MH144564–MH144569. In total, six SNPs were identified (Fig. 1). There was only one SNP in the exon 3 coding region, which was a synonymous SNP c.153T>C. The frequencies of these sequences in the NZ Romney sheep investigated are illustrated in Figure 1.

### Effect of sequence variation in IGF1 on carcass and growth traits

In the 5′ flanking region one sequence (*B_1_*; c.-648C and c.-646A) was predominant, whereas the other sequence (*A_1_*; c.-648G and c.-646G) occurred at a frequency of <5%, hence the association analyses were only undertaken for the exon 3 and exon 4 regions.

For the exon 3 region, an effect of the presence/absence of PCR-SSCP variant was observed for growth rate to weaning (Table 2), with the presence of *B_2_* being associated (*p* = 0.048) with a lower growth rate (present: 384.9 ± 3.77 grams/day; absent: 396.0 ± 5.44 grams/day). An effect of the presence/absence of PCR-SSCP variant was also observed for HCW, V-GR, and proportion leg yield (Table 2), with the presence of *B_2_* being associated with increased HCW (*p* = 0.015) and increased V-GR (*p* = 0.003), but decreased proportion leg yield (*p* = 0.012). For this exon 3 region, an effect of genotype was observed for HCW, V-GR, and shoulder yield (Table 3). Sheep with genotype *B_2_B_2_* (c.64-82CC and c.153CC) had lower HCW (*p* = 0.010), lower V-GR (*p* = 0.005) and less shoulder yield (*p* = 0.021) than those sheep of genotype *A_2_A_2_* (c.64-82AA and c.153TT) or *A_2_B_2_* (c.64-82AC and c.153TC).

**Table 2.** Association of *IGF1* Exon 3 Sequences with Growth and Carcass Traits in New Zealand Romney Sheep

Trait^a^	Variant	n		Mean ± SE^b^		p
		Absent	Present	Absent	Present	
Birth weight (kg)	*A_2_*	209	506	5.68 ± 0.07	5.77 ± 0.05	0.255
	*B_2_*	144	571	5.78 ± 0.08	5.74 ± 0.05	0.659
Growth rate to weaning (grams/day)	*A_2_*	209	506	390.1 ± 5.13	387.0 ± 3.68	0.532
	*B_2_*	144	571	**396.0 ± 5.44**	**384.9 ± 3.77**	**0.048**
HCW (kg)	*A_2_*	127	316	**16.74 ± 0.23**	**17.21 ± 0.20**	**0.015**
	*B_2_*	93	350	17.22 ± 0.25	17.02 ± 0.20	0.350
V-GR (mm)	*A_2_*	127	316	**7.04 ± 0.32**	**7.84 ± 0.27**	**0.003**
	*B_2_*	93	350	7.82 ± 0.34	7.52 ± 0.27	0.316
Shoulder yield (%)	*A_2_*	127	316	*17.01 ± 0.10*	*17.18 ± 0.09*	*0.052*
	*B_2_*	93	350	17.13 ± 0.11	17.12 ± 0.09	0.974
Loin yield (%)	*A_2_*	127	316	14.83 ± 0.10	14.92 ± 0.09	0.316
	*B_2_*	93	350	14.90 ± 0.11	14.90 ± 0.09	0.869
Leg yield (%)	*A_2_*	127	316	22.19 ± 0.14	22.13 ± 0.12	0.591
	*B_2_*	93	350	22.17 ± 0.15	22.15 ± 0.12	0.857
Total yield (%)	*A_2_*	127	316	54.03 ± 0.29	54.22 ± 0.24	0.415
	*B_2_*	93	350	54.20 ± 0.31	54.15 ± 0.24	0.874
Proportion shoulder yield (%)	*A_2_*	127	316	*31.49 ± 0.13*	*31.68 ± 0.11*	*0.080*
	*B_2_*	93	350	31.61 ± 0.14	31.63 ± 0.11	0.843
Proportion loin yield (%)	*A_2_*	127	316	27.44 ± 0.11	27.50 ± 0.09	0.505
	*B_2_*	93	350	27.49 ± 0.11	27.48 ± 0.09	0.918
Proportion leg yield (%)	*A_2_*	127	316	**41.06 ± 0.12**	**40.81 ± 0.10**	**0.012**
	*B_2_*	93	350	40.90 ± 0.13	40.89 ± 0.10	0.900

^a^ HCW—hot carcass weight; V-GR—VIAscan fat depth at the 12th rib.

^b^ Predicted means and standard error of those means derived from GLMMs, with various factors being included in the models for different traits as described in the Materials and Methods section. *p* < 0.05 are in bold, whereas 0.05 ≤ *p* < 0.10 are italicized.

GLMMs, General Linear Mixed-Effects Models.

**Table 3.** Association of *IGF1* Exon 3 Genotypes with Growth and Carcass Traits in New Zealand Romney Sheep

Trait^*^	Mean ± SE^**^			p
	A_2_A_2_	A_2_B_2_	B_2_B_2_	
	(*n* = *144*)	(*n* = *362*)	(*n* = *209*)	
Birth weight (kg)	5.78 ± 0.08	5.76 ± 0.05	5.68 ± 0.07	0.515
Growth rate to weaning (grams/day)	395.07 ± 5.08	385.96 ± 3.54	388.76 ± 4.55	0.256
	(*n* = 93)	(*n* = 223)	(*n* = 127)	
HCW (kg)	**17.31 ± 0.25^a^**	**17.17 ± 0.20^a^**	**16.63 ± 0.23^b^**	**0.010**
V-GR (mm)	**7.91 ± 0.34^a^**	**7.67 ± 0.28^a^**	**6.88 ± 0.31^b^**	**0.005**
Shoulder yield (%)	**17.24 ± 0.10^a^**	**17.24 ± 0.08^a^**	**17.02 ± 0.09^b^**	**0.021**
Loin yield (%)	14.91 ± 0.11	14.92 ± 0.09	14.83 ± 0.11	0.604
Leg yield (%)	22.16 ± 0.15	22.12 ± 0.13	22.19 ± 0.14	0.824
Total yield (%)	54.22 ± 0.24	54.30 ± 0.23	53.91 ± 0.52	0.717
Proportion shoulder yield (%)	31.79 ± 0.12	31.83 ± 0.11	31.62 ± 0.12	0.148
Proportion loin yield (%)	27.46 ± 0.10	27.38 ± 0.08	27.35 ± 0.10	0.648
Proportion leg yield (%)	40.76 ± 0.11	40.79 ± 0.10	40.97 ± 0.11	0.126

^*^ HCW—hot carcass weight; V-GR—VIAScan fat depth at the 12th rib.

^**^ Predicted means and standard error of those means derived from the GLMMs, with means that do not share a superscript letter (a or b) within rows being different at *p* < 0.05 and shown in bold.

No associations were detected for the exon 4 region (results not shown).

## Discussion

This is the first report describing associations between sequence variation in ovine *IGF1* and growth and carcass traits in NZ Romney lambs. There was only a single synonymous SNP detected in the coding region of *IGF1*, which is in agreement with the observation that *IGF1* is conserved among mammals and that the IGF1 protein, along with IGF2 and insulin, comprise a conserved protein family found in most mammalian species and in many other vertebrates (Rotwein, 2017). Highly conserved sequences are typically associated with proteins that underpin conserved or essential metabolic activities (Zhao *et al.*, 2018), and mice that are *IGF1*-null (created by homologous recombination), exhibit postnatal lethality, growth retardation, infertility, and profound defects in the development of their major organ systems, with this confirming the essential nature of the protein's activity (Liu *et al.*, 2000).

The effect of SNPs in the 5′ flanking region cannot be reliably assessed in this study due to the minor sequence *A* (c.-648G and c.-646G) occurring at a low frequency (4.4%) in the NZ Romney sheep investigated. However, the sequence frequencies in this region appear to be interesting. In Iranian Zandi sheep, a medium-sized dual-purpose breed used for meat and pelt production and found in the central region of Iran, those with the nucleotide sequence variation that was also revealed in *A* (c.-648G and c.-646G) constituted 47% of the population (Nazari *et al.*, 2016). In Colored Polish Merino sheep, c.-648G and c.-646G are common, with a frequency of 91.6% reported (Grochowska *et al.*, 2017) and it is detected at a frequency of 19.1% in Small Tail Han sheep (primarily a meat breed in China), and was very rare or absent in Texel and Dorset sheep (both meat breeds) (He *et al.*, 2012). Whether this difference in sequence frequency is related to meat/wool/pelt production, or just reflects breed differences, awaits further investigation. However, the findings that the SNPs in this region affected wool production, with *A* (c.-648G and c.-646G) being associated with increased clean fleece weight in Egyptian Barki sheep (Darwish *et al.*, 2017) and that *IGF1* transgenic sheep produced more clean fleece than their nontransgenic half-sibs at yearling shearing (Damak *et al.*, 1996), suggest that *IGF1* may play a role in regulating wool growth and production.

The finding of associations between polymorphism in *IGF1* and growth traits is notable. The two SNPs in the 5′ flanking region described in Ramasamy (2018), Nazari *et al.* (2016), Grochowska *et al.* (2017), and in this study, were associated with growth traits in Baluchi sheep (*n* = 102; Tahmoorespur *et al.*, 2009), Makui sheep (*n* = 100; Hajihosseinlo *et al.*, 2013), and Makooei sheep (number unknown; Negahdary *et al.*, 2013). In addition, Trukhachev *et al.* (2016) found associations between the SNPs c.-5363C>T, c.-5188G>C, c.-5186G>A and c.-4088G>A, and live weight, and reported that c.-91A>C had a correlation with live weight, wither height, croup height, width and length, and other physical attributes in rams. Associations with the 5′ flanking SNPs could not be tested in this study. However Proskura and Szewczuk (2014) and Gholibeikifard *et al.* (2013), investigated the effect of X69473.1:g271C>T (equivalent to c.153C>T in this study) in Pomeranian Coarse-wool sheep and Baluchi sheep, respectively, and did not find any association with growth traits. Ramasamy (2018), Nazari *et al.* (2016) and Grochowska *et al.* (2017) investigated polymorphism in the 5′ flanking region of *IGF1* and also did not detect any association with growth traits in different sheep breeds.

It is unknown whether the effect of *IGF1* polymorphism on growth traits is breed dependent, but given some of the associations were typically detected with small numbers of sheep and/or there was the lack of genetic background for statistical correction, caution should be taken when interpreting these results, and further investigations may be required to confirm the findings.

The associations detected for the exon 3 PCR-SSCP variants and HCW, V-GR, and shoulder yield suggest that exon 3 nucleotide sequence variation affects selected carcass traits, although Trukhachev *et al.* (2016) revealed no associations between the synonymous substitution of c.81T>C in this exon and meat production parameters.

Given that HCW and V-GR have a moderate positive correlation (*r* = 0.573; Supplementary Table S1), the associations detected for HCW and V-GR may be due to these traits being correlated. Polymorphism in the 5′ flanking region of *IGF1* affected EUROP fat class, kidney fat class, and external fatness of carcass class in Colored Polish Merino sheep (Grochowska *et al.*, 2017). Another study in Mehraban sheep describes how *IGF1* polymorphism is associated with the triglyceride and cholesterol content of blood and the authors reported a tendency for association of the *IGF1* polymorphism with dorsal fat thickness (Behzadi *et al.*, 2015). In cattle, a *Sna*BI polymorphism in the regulatory region of the *IGFI* associated with subcutaneous back fat (Curi *et al.*, 2005), and a promoter SNP in *IGF1* associated with ultrasound back fat thickness and carcass average back fat in the Angus beef (Islam *et al.*, 2009). With transgenic mice, IGFI has been shown to be involved in fat cell development (Rajkumar *et al.*, 1999). The findings of this study and others suggest that *IGFI* could be considered as a candidate gene for fat-related carcass traits.

Shoulder yield only had a weak correlation with both HCW and V-GR, suggesting that whatever effect the *IGF1* polymorphism was having, it may be different to how it might affect V-GR or HCW. The effect of *IGF1* polymorphism on meat yield has been reported for both sheep and beef cattle, with Grochowska *et al.* (2017) describing how 5′ flanking region polymorphism affects fore shank weight, although they did not reveal an effect on shoulder yield in the Colored Polish Merino sheep. A promoter SNP associated with carcass lean meat yield in the Angus beef population, but not in Charolais cattle and hybrid Charolais × Angus cattle (Islam *et al.*, 2009). This suggests that different SNPs in *IGF1* may have different effects on meat yield and/or the effect may vary between breeds.

The IGF1 gene is located on ovine chromosome 3, which to date has had at least 60 quantitative trait loci (QTL) located on it (sheep QTL database, 2019), including markers for birth weight, body weight, muscle depth, and subcutaneous fat thickness.

The genotype associations detected for HCW, V-GR, and shoulder yield suggest that *B_2_* is associated with a decrease in HCW, V-GR, and shoulder yield, whereas *A_2_* (c.64-82A and c.153T) is associated with an increase in HCW, V-GR, and shoulder yield. As there was no difference in the marginal means for these traits between *A_2_A_2_* (c.64-82AA and c.153TT) and *A_2_B_2_* (c.64-82AC and c.153TC) sheep, this suggests that *B_2_* (c.64-82C and c.153C) has a recessive effect, whereas *A_2_* (c.64-82A and c.153T) has a dominant effect on these traits.

The effect of *A_2_* (c.64-82A and c.153T) may come about directly as a result of the two SNPs. Although the SNP in the coding region (c.153T>C) was synonymous, and would not result in an amino acid substitution, it may affect the expression or structure of the protein. It has been reported that silent mutations may affect mRNA translation rates and thus potentially change the way that protein folds (Hurst, 2011). With the intronic SNP c.64-82A>C, introns are known to carry regulatory sequences, so although they may not have a direct involvement in the regulation of transcription of highly expressed genes (Mullen *et al.*, 2011), they can affect alternative splicing mechanism and may be associated with mRNA transport or chromatin assembly (Jo and Choi, 2015).

Finally, it is quite possible that the effects observed in this research are due to the SNPs observed being linked to nucleotide sequence variation in other regions of the gene that regulate gene expression or function.

## Conclusions

This study used PCR-SSCP to screen for nucleotide sequence variation in the 5′ flanking region, exon 3, and exon 4 regions of ovine *IGF1*. Six previously identified SNPs were identified in 848 NZ Romney sheep. In different models, sequence variation in exon 3 of *IGF1* was associated with growth rate to weaning, HCW, V-GR, and shoulder lean meat yield and proportion leg yield. Verification of these findings will require further testing in more sheep from different flocks and breeds.

## Supplementary Material

Supplemental data
