# Supplementary material for: Nucleotide Sequence Variation in the Insulin-Like Growth Factor 1 Gene Affects Growth and Carcass Traits in New Zealand Romney Sheep
Source: DNA Cell Biol. 2021 Feb 11;40(2):265–71. doi: 10.1089/dna.2020.6166 (PMC7891192; doi:10.1089/dna.2020.6166)
Supplement: Supplemental data [file Supp_TableS1.docx]

**Supplementary Table S1. Correlations between growth and carcass traits**

|  | Birth weight | Growth rate to weaning | HCW^1^ | V-GR | Leg yield | Loin yield | Shoulder yield | Total yield | Proportion leg yield | Proportion loin yield |
| --- | --- | --- | --- | --- | --- | --- | --- | --- | --- | --- |
| Growth rate to weaning | 0.036 |  |  |  |  |  |  |  |  |  |
| HCW | 0.309*** | 0.014 |  |  |  |  |  |  |  |  |
| V-GR | 0.159** | 0.010 | 0.573*** |  |  |  |  |  |  |  |
| Leg yield | -0.038 | 0.000 | -0.176*** | -0.559*** |  |  |  |  |  |  |
| Loin yield | 0.024 | -0.052 | 0.220*** | -0.174*** | 0.681*** |  |  |  |  |  |
| Shoulder yield | 0.159** | -0.007 | 0.233*** | 0.043 | 0.422*** | 0.434*** |  |  |  |  |
| Total yield | 0.051 | -0.021 | 0.083 | -0.312*** | **0.880***** | **0.846***** | **0.735***** |  |  |  |
| Proportion leg yield | -0.173*** | 0.036 | -0.518*** | -0.613*** | 0.520*** | -0.088 | -0.433*** | 0.053 |  |  |
| Proportion loin yield | -0.028 | -0.064 | 0.289*** | 0.125** | 0.007 | 0.647*** | -0.246*** | 0.140** | -0.239*** |  |
| Proportion shoulder yield | 0.172*** | 0.016 | 0.237*** | 0.441*** | -0.458*** | -0.403*** | 0.559*** | -0.150** | -0.694*** | -0.533*** |

^1^HCW = Hot carcass weight. Correlations with |r| > 0.7 are in bold, and those with 0.3 < |r| ≤ 0.7 are underlined. ***P* < 0.01; ****P* < 0.001.
